# Pelvic Hematoma After UroLift: A Case Report and Literature Review

**DOI:** 10.7759/cureus.38193

**Published:** 2023-04-27

**Authors:** Laura F Rodríguez-Fernández, Claudio P Bernaschina-Bobadilla

**Affiliations:** 1 Surgery, Ponce Health Sciences University, Ponce, PRI; 2 Urology, St. Luke's Episcopal Hospital, Ponce, PRI

**Keywords:** urolift, urologic surgery, benign prostatic hyperplasia, surgery, pelvic hematoma after urolift, pelvic hematoma, complication of urolift, urology

## Abstract

UroLift is a novel, minimally invasive surgical technique used to treat bladder outlet obstruction due to benign prostatic hyperplasia (BPH). UroLift was granted US FDA approval in 2013, and so far, it has gained acceptance and popularity worldwide. In this case report, we present a 69-year-old male patient that developed a pelvic hematoma with subacute clinical manifestations two months following UroLift. The patient was managed conservatively, resulting in the complete resolution of the hematoma. As more surgeons are trained, and the caseload increases, we expect to see more complications related to this novel technique. Surgeons should be aware of this procedure's potential short- and long-term complications.

## Introduction

Benign prostatic hyperplasia (BPH) is seen with increased prevalence in men of advancing age [1]. It is estimated that in the United States, 15 million men over 30 years old have the condition or have lower urinary tract symptoms (LUTS) [2]. In patients over the age of 50, approximately 50%-75% have BPH, and for those over 70 years old, 80% live with the condition [2]. BPH is characterized histologically by non-malignant hyperplasia that occurs due to stromal and epithelial cell proliferation in the transition zone of the prostate [1]. This hyperplasia causes a benign enlargement of the glandular tissue surrounding the prostatic urethra, leading to bladder outlet obstruction and subsequent LUTS [1]. These symptoms can include incomplete emptying of the bladder, urinary retention, frequency, urgency, incontinence, sexual dysfunction, nocturia, and UTIs [1,3]. Treatment for BPH will depend on the severity of the patient's symptoms [1]. Treatment options include observation with lifestyle changes, medical therapy, or a surgical approach. Medical therapy includes alpha-blockers and 5-alpha reductase inhibitors. Alpha-blockers relax the stromal smooth muscle of the prostatic urethra and the bladder neck, reducing resistance and improving the urinary flow [1]. The 5-alpha reductase inhibitors prevent the conversion of testosterone to dihydrotestosterone, the active metabolite [1]. This reduces the hormonal stimulation of the stromal tissue, leading to a subsequent decrease in prostatic volume [1]. Surgical approaches include transurethral resection of the prostate (TURP), laser photoselective vaporization of the prostate (LVP), and Holmium laser enucleation of the prostate (HoLEP) [1]. The minimally invasive therapies available are transurethral water vapor therapy (Rezum) and prostatic urethral lift (UroLift) [1]. UroLift is a minimally invasive tissue-sparing approach that widens the prostatic urethra using small implants that compress the lateral prostatic lobes [1]. The US FDA granted approval for UroLift 10 years ago. UroLift has gained worldwide popularity due to the favorable surgical outcomes, decreased recovery time, and diminished side effects compared to other surgical approaches [3]. So far, worldwide, more than 400,000 patients have been treated with UroLift [4]. Several studies have been performed to evaluate the outcomes of patients after UroLift placement [3]. Results of these studies show that most patients have marked improvement in quality of life and the International Prostate Symptom Score (IPSS), reduction in LUTS, and no deficiencies in erectile or ejaculatory function [3]. Most side effects following UroLift placement are transient in nature and self-limited. They should resolve by approximately one month after the procedure. These symptoms might include irritative symptoms, incontinence, hematuria, dysuria, and pelvic pain [3]. Although rare, some major adverse effects, such as the formation of pelvic hematomas and encrustation of implants inserted too proximally to the bladder [3], have been reported in the literature with UroLift.

## Case presentation

**Figure 1:**
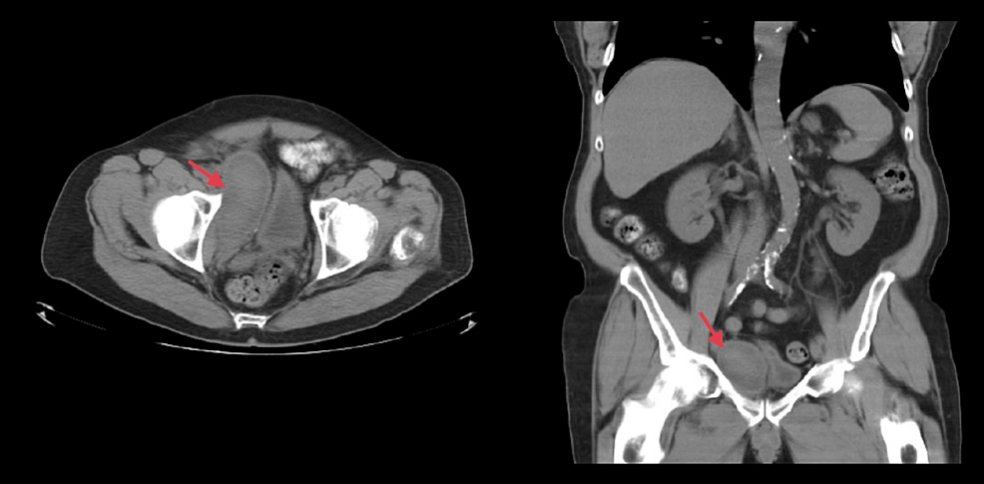
Pelvic CT scan eight weeks after UroLift showing a right pelvic soft tissue density mass with central low attenuation measuring 10.4 cm x 4.5 cm x 5.0 cm in diameter exerting a mass effect on the urinary bladder consistent with a pelvic hematoma.

We present the case of a 69-year-old male with a medical history of hypertension, diabetes mellitus, and culture-proven UTIs. He was referred to the urologist due to BPH, LUTS, and UTIs. The patient had been using tamsulosin for more than five years. He had an IPSS of four; scores of 0-7 are remarkable for mild symptoms, 8-19 for moderate symptoms, and 20-35 for severe symptoms [5]. A digital rectal examination showed a large rubbery gland with no hard nodules. A post-void residual (PVR) amount of urine revealed 150 mL (<50 mL is adequate, in the elderly, 50-100 mL can be considered normal). His baseline prostate-specific antigen (PSA) was 3.14 ng/dL, where usually less than 4.0 ng/dL is considered within normal limits. Transrectal ultrasound showed a prostatic volume of 42 grams (normal prostate volume is around 20-25 grams), and cystoscopy showed obstructive lateral lobes of the prostate. The patient had compliance issues and failed drug therapy; therefore, he was offered to restart medical management or consider surgical options, so he opted for UroLift. The main reasons for the intervention were recurrent UTIs and increased PVR. The patient had an uneventful UroLift procedure performed on April 1, 2022. Four implants were placed, and the patient went home without a urinary catheter. There were no intraoperative or immediate postoperative complications. Two weeks after UroLift, the patient was seen in the office. He complained only of irritative symptoms, PVR decreased to 40 mL, but IPSS increased to 21. At the six-week follow-up, the patient no longer had irritative symptoms and had a stronger urinary stream but experienced an episode of prostatitis that resolved with antibiotic treatment. His PSA rose to 19 ng/dL. Eight weeks after UroLift, the patient visited his primary care physician (PCP) complaining of bothersome low pelvic pain. His PCP requested a double contrast pelvic CT scan. The CT scan revealed a right-sided pelvic soft tissue density mass with central low attenuation measuring 10.4 cm x 4.5 cm x 5.0 cm in diameter that was exerting a mass effect on the urinary bladder. It was consistent with a pelvic hematoma (Figure 1). At this visit, the patient's vital signs were within normal limits; hemoglobin was 12.39 g/dL (pre-procedure hemoglobin was 13.97 g/dL), PSA had decreased to 4.12 ng/dL, and had a PVR of 14 mL. Thus a decision for conservative management of the hematoma was taken.

**Figure 2:**
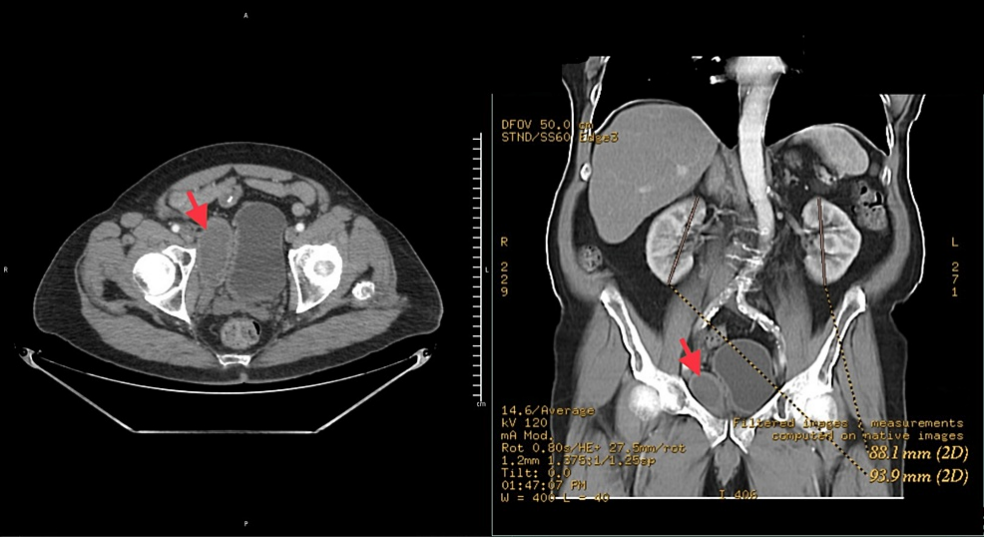
Pelvic CT scan 12 weeks after UroLift showing a 6.8 cm x 3.4 cm mass resolving right-sided pelvic hematoma.

Follow-up pelvic CT scan 12 weeks after UroLift showed a 6.8 cm x 3.4 cm mass resolving right-sided pelvic hematoma (Figure 2). The patient again had stable vital signs, the LUTS were improving, and he had a stronger urinary stream.

**Figure 3:**
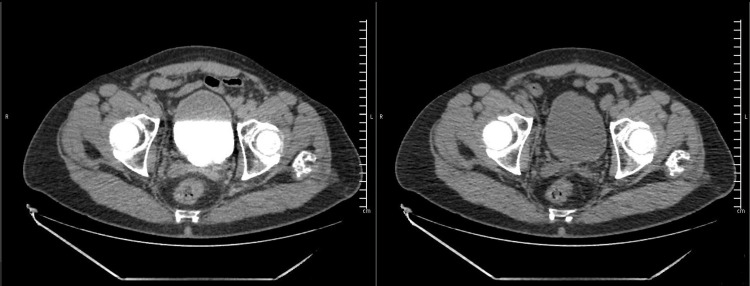
Pelvic CT scan confirming the complete resolution of the pelvic hematoma.

At the seventh month visit after UroLift, the patient stated that he was voiding well, no longer had any complaints about pelvic pain or discomfort, had a PVR of 30 mL, and an IPSS of 10. Nine months after UroLift, and seven months after the diagnosis of the hematoma, a pelvic CT scan confirmed the complete resolution of the pelvic hematoma (Figure 3).

The management of this patient included conservative measures and continuous monitoring of his clinical condition. The pelvic hematoma was able to undergo resorption and full resolution without requiring medical or surgical interventions. At the 12-month postoperative visit, the patient had no complaints, a PVR of 30 mL, and an IPSS of two (Table 1).

**Table 1:** Patient's results before and after UroLift.

Values	Patient’s results before UroLift	Patient’s results after UroLift	Reference ranges
International Prostate Symptom Score (IPSS)	4	2 (12 months after)	0-7 (mild symptoms), 8-19 (moderate symptoms), 20-35 (severe symptoms) [5]
Post-void residual (PVR) volume	150 mL	30 mL (12 months after)	<50 mL in adults is adequate, in elderly 50-100 mL can be considered normal
Prostate-specific antigen (PSA)	3.14 ng/dL	4.12 ng/dL (eight weeks after)	< 4.0 ng/dL is within normal limits
Prostatic volume	42 grams	-	20-25 grams is normal

## Discussion

UroLift is a relatively new minimally invasive surgical procedure that has been gaining popularity during the past few years [3]. So far, around 400,000 cases have been performed worldwide [4]. Past literature reporting complications and adverse effects after the UroLift procedure is scarce. To our knowledge, there are four case reports published in the literature describing the development and management of pelvic hematomas after the placement of UroLift implants [6-9]. The first appearance of this complication was reported in 2019, where Pollock GR et al. reported the case of a patient that presented with pelvic pain, anemia, and penile ecchymosis and edema after UroLift [6]. The patient subsequently underwent imaging studies that demonstrated the development of bilateral pelvic hematomas on postoperative day four that were conservatively managed [6]. The second published case was by Cai PY et al., and it regards a patient that developed refractory hypotension, abdominal pain, and urinary retention in the recovery room after UroLift [7]. CT scanning in this patient revealed a large pelvic hematoma [7]. The patient showed signs of worsening clinical condition with increasing abdominal pain, decreasing hemoglobin, elevation of lactate levels, and hypotension despite intravenous fluids (IVFs), vasopressors, and blood transfusions [7]. Consequently, the patient underwent emergency exploratory laparotomy, which uncovered a small active bleeding vessel on the left pubic bone that was the cause of the pelvic hematoma; ligation of the vessel controlled the bleeding, and the hematoma was drained [7]. Ewing B et al. published the third case concerning the development of a pelvic hematoma after UroLift [8]. This patient presented with syncope in the recovery room after removing the urinary catheter, he was managed with IVFs and bed rest for two hours, but upon standing, the patient experienced another syncope and hypotension [8]. The patient was eventually transferred to the ED, where evaluation confirmed hypotension, abdominal distention, and decreasing hemoglobin, and CT imaging showed a large pelvic hematoma [8]. The management that this patient received consisted of the transfusion of 10 units of packed RBCs and hemodialysis for renal failure for one week [8]. This case introduces a distinctive presentation because the patient had a baseline stage III chronic kidney disease (CKD) that evolved to stage IV CKD; this was due to acute blood loss that resulted in prolonged hypotension and contrast from imaging, which resulted in a superimposed acute kidney injury [8]. The fourth reported case was by Roehmholdt MJ and Bentley DF, and it consisted of a patient who had an uneventful UroLift procedure that presented 16 hours later to the ED with abdominal pain [9]. Initial management performed comprised IVFs and CT scanning, which revealed a large pelvic extraperitoneal hemorrhage [9]. The patient was followed for leukocytosis and decreasing hemoglobin; additional imaging studies included a pelvic CT scan with a cystogram which was negative for extravasation [9]. The patient had a decreasing leukocytosis, but blood work also revealed decreasing hemoglobin which led to the transfusion of three units of packed RBCs [9]. With this management, the patient was clinically stable and did not require additional treatment; at the two-week follow-up, the patient presented left flank, penile and scrotal ecchymosis but no other symptomatology [9].

The distinctiveness and uniqueness of our case report is the subacute presentation of the pelvic hematoma. This patient's symptoms were prominent eight weeks after the UroLift procedure, which differs from previously reported cases that all presented with acute symptoms. Additionally, this patient did not require blood transfusions or surgical interventions, as conservative management resulted in the resolution of the pelvic hematoma. As stated by Roehmholdt MJ and Bentley DF, there might be a potential for implants going beyond the prostatic tissue resulting in injury to a vessel [9]; therefore, measuring prostatic volume before the procedure is essential to be wary about possible complications. The indolent formation and clinical presentation of this patient's pelvic hematoma could lead us to believe there might have been a venous injury during the procedure, as opposed to an arterial injury due to the delayed presentation. As seen in the previous case reports, the clinical manifestations of pelvic hematomas after UroLift can have varied clinical presentations and, ultimately, different clinical management. This highlights the importance of taking proper informed consent and discussing with our patients all the possible complications that can happen after UroLift, such as transient adverse events, which include dysuria, hematuria, and pelvic pain [3]. In addition, complications reported such as mild-to-moderate Clavien-Dindo Grade 1 events, the need for retreatment with surgical or medical approaches, the removal of encrusted implants, implants susceptible to encrustation such as those inserted too proximally with possible intrusion into the urinary bladder, formation of pelvic hematomas, and two complications reported not related to the implants that were clot retention with restarting warfarin and bladder stone formation, or groin pain due to a mistakenly placed implant that resulted in ureteric obstruction with hydronephrosis and calyceal rupture [3]. Conversation of such risks should include the discussion of possible pelvic hematomas despite it being a rare adverse effect.

## Conclusions

As more patients are treated with UroLift for BPH, we expect to see a rise in the occurrence and reporting of adverse outcomes and complications, such as the pelvic hematoma we are reporting. Physicians should be encouraged to report complications seen in the acute period, but also long-term complications. As UroLift gains more popularity, clinicians should be aware of the complications and suspect them in patients with pelvic pain during the postoperative period that does not resolve in the usual one to two weeks. As previously stated, management will depend on the patient's presentation and clinical condition. It can range from conservative management to emergency surgical interventions with requirements for blood transfusions.
